# Luminal and basal-like breast cancer cells show increased migration induced by hypoxia, mediated by an autocrine mechanism

**DOI:** 10.1186/1471-2407-11-158

**Published:** 2011-05-02

**Authors:** Melanie J Voss, Mischa F Möller, Desmond G Powe, Bernd Niggemann, Kurt S Zänker, Frank Entschladen

**Affiliations:** 1 Institute of Immunology, ZBAF, Witten/Herdecke University, Stockumer Str. 10, 58448 Witten, Germany; 2 Department of Cellular Pathology, Queen's Medical Centre, Nottingham University Hospitals NHS Trust, Nottingham, NG7 2UH, UK

## Abstract

**Background:**

Some breast cancer patients receiving anti-angiogenic treatment show increased metastases, possibly as a result of induced hypoxia. The effect of hypoxia on tumor cell migration was assessed in selected luminal, post-EMT and basal-like breast carcinoma cell lines.

**Methods:**

Migration was assessed in luminal (MCF-7), post-EMT (MDA-MB-231, MDA-MB-435S), and basal-like (MDA-MB-468) human breast carcinoma cell lines under normal and oxygen-deprived conditions, using a collagen-based assay. Cell proliferation was determined, secreted cytokine and chemokine levels were measured using flow-cytometry and a bead-based immunoassay, and the hypoxic genes HIF-1α and CA IX were assessed using PCR. The functional effect of tumor-cell conditioned medium on the migration of neutrophil granulocytes (NG) was tested.

**Results:**

Hypoxia caused increased migratory activity but not proliferation in all tumor cell lines, involving the release and autocrine action of soluble mediators. Conditioned medium (CM) from hypoxic cells induced migration in normoxic cells. Hypoxia changed the profile of released inflammatory mediators according to cell type. Interleukin-8 was produced only by post-EMT and basal-like cell lines, regardless of hypoxia. MCP-1 was produced by MDA-MB-435 and -468 cells, whereas IL-6 was present only in MDA-MB-231. IL-2, TNF-α, and NGF production was stimulated by hypoxia in MCF-7 cells. CM from normoxic and hypoxic MDA-MB-231 and MDA-MB-435S cells and hypoxic MCF-7 cells, but not MDA-MB-468, induced NG migration.

**Conclusions:**

Hypoxia increases migration by the autocrine action of released signal substances in selected luminal and basal-like breast carcinoma cell lines which might explain why anti-angiogenic treatment can worsen clinical outcome in some patients.

## Background

One of the hallmarks of cancer is the replicative potential of tumor cells [1]. However, fast growing tumors need to be supplied with nutrients and oxygen which cannot be sufficiently sustained by diffusion alone and so requires sustained angiogenesis [1]. Without angiogenesis, oxygen deprivation occurs, and to evade this fate, hypoxic tumor cells release cell signalling substances that induce angiogenesis [2], regulated by hypoxia-inducible factor (HIF) which is a key element in the hypoxic pathway [3]. Therapeutic anti-angiogenic strategies have been established to limit tumour growth [4] and because of its pivotal role HIF-1α is especially targeted for such treatment [5]. HIF-1α over-expression and signalling are reported to correlate with poor prognosis and high metastasis formation [6,7]. Paradoxically, therapeutic anti-angiogenic or angiostatic strategies have been proposed to increase in metastasis formation [6,8].

Basal-like breast cancers differ to luminal cancers in being triple negative for the immunophenotypic markers ER^-^/PGR^-^/HER2^- ^but express CK5/6 [9] and in addition, they show increased hypoxia and high tumor grade [10,11]. Consequently, basal-like cancers have an aggressive phenotype characterized by high cell proliferation and poor clinical outcome but contrary to expectations, we recently showed that these tumors do not always show increased metastasis [12]. Most breast cancer associated deaths are due to metastatic spread into distant organs. This dissemination of tumor cells can occur early in the cancer disease and often remains initially undetected [13]. Cell migration is a prerequisite for metastasis formation and we have shown previously that several neurotransmitters induce migration of MDA-MB-468 human breast carcinoma cells, with dopamine and norepinephrine having the strongest effects [14]. In these cells, the enhanced migratory activity in response to norepinephrine is based on the activation of the motor protein non-muscle myosin II [15], and is accompanied by changes in gene expression towards a metastatogenic phenotype [16]. Similarly, chemokines and cytokines are released in the tumor environment by the tumor cells themselves as well as leukocytes, fibroblasts and other cells of the tumor stroma. Therefore, tumors are often compared with non-healing wounds, and these inflammatory mediators are supposed to support tumor progression with regard to metastasis formation [17,18]. Previous studies have shown that hypoxia can induce basal-like and epithelial-to-mesenchymal transition (EMT) properties in breast cancer [19]. In the current study, we hypothesised that hypoxia induces cell migration in breast cancer and that this is achieved through the involvement of inflammatory cell mediators. We investigated the migratory activity of luminal (MCF-7), post-EMT (MDA-MB-231, MDA-MB-435S), and basal-like (MDA-MB-468) human breast carcinoma cell lines under normal and oxygen-deprived conditions and the secretion of inflammatory cytokines and chemokines.

## Methods

### Breast cancer cell lines

We used four human breast carcinoma cell lines for all experiments, selected for being representative of the luminal-like (oestrogen receptor (ER) positive) and basal-like molecular classes described in breast cancer gene expression studies [20-25] (Table 1). MCF-7 is a well characterised ER-positive (luminal-like) cell line. MDA-MB-231 and MDA-MB-435S share basal-like properties and are classified as post-epithelial-mesenchymal transition (post-EMT) cells due to vimentin positivity [26,27]. MDA-MB-468 is a basal-like cell line expressing cytokeratin (CK) 5/6 [28,29].

**Table 1:** Characterisation of the investigated breast cancer cell lines

Cell line	ER	PGR	HER-2	EGFR	CK5/6	Vimentin
MDA-MB-231	negative	negative	normal	positive	negative	positive
MDA-MB-435S	negative	negative	amplified	negative	negative	positive
MDA-MB468	negative	negative	normal	positive	positive	negative
MCF-7	positive	positive	normal	negative	negative	negative

The cell line MCF-7 was derived from the German Collection of Microorganisms and Cell Cultures (DSMZ, Braunschweig, Germany), the cell lines MDA-MB-231, MDA-MB-435S and MDA-MB-468 were derived from the American Type Culture Collection (ATCC, Manassas, VA). The three MDA-MB cell lines were cultured in DMEM medium (PAA, Pasching, Austria) containing 10% heat-inactivated fetal calf serum (PAA), and the MCF-7 cells were cultured in RPMI medium (PAA) containing 10% heat-inactivated fetal calf serum and 1% penicillin/streptomycin solution (50 U/ml and 50 μg/ml, respectively; GIBCO, Eggenstein-Leopoldshafen, Germany), in addition supplemented with non-essential amino acids (PAA), 1 mM sodium pyruvate (Biochrom AG, Berlin, Germany) and 10 μg/ml insulin (Sigma-Aldrich, Taufkirchen, Germany). All cultures were kept at 37°C in a humidified atmosphere containing 5% CO_2_. Hypoxia was induced by a stepwise oxygen-deprivation in hypoxia-chambers (Billups-Rothenberg, Del Mar, CA). The cells were kept for two days at 10% O_2_, for one day at 5% O_2_, and for one day at 1% O_2 _with constantly 5% CO_2 _and the remainder being N_2_. It is worth to note that there is currently no consistent standard protocol for the *in vitro *induction of hypoxia in tumor cells.

### Isolation of neutrophil granulocytes

Human neutrophil granulocytes (NG) were isolated from peripheral blood of voluntary healthy donors by a two-step protocol as described previously [15]. Informed consent was obtained from all donors according to the Declaration of Helsinki. The origin of the blood was blinded and made anonymous according to ethic requirements. The heparinized blood was diluted with PBS (1:1.7). Neutrophil granulocytes together with erythrocytes were separated from the peripheral blood mononuclear cell fraction by density gradient centrifugation on lymphocyte separation medium (LSM 1077; PAA, Pasching, Austria). Subsequently, NG were isolated by mixing platelet-depleted serum from the same blood donor, diluted 1:1.3 with a high molecular weight dextran solution (Macrodex; Fresenius, Bad Homburg, Germany) containing 0.01 M EDTA. After 3 hours, the supernatant containing granulocytes was isolated and any remaining erythrocytes were removed by hypotonic lysis with 0.3% sodium chloride on ice. NG were used for experiments immediately after isolation.

### Cell migration assay

The three-dimensional collagen-based migration assay was performed as described previously [15]. In brief, 8 × 10^4 ^tumor cells or 2.5 × 10^5 ^NG were resuspended in 50 μl of normal culture medium without any supplements, or in culture medium conditioned by either normoxic or hypoxic tumor cells. Conditioned medium was prepared as follows: 500,000 cells of each cell line were seeded in 4 ml full culture medium and cultured under normoxic or hypoxic conditions as described above. After three days (at the time when the oxygen content was reduced from five to one percent concerning the hypoxic cells) the regular culture media was removed and replaced by 1 ml of the media without supplements. After incubation for 24 hours at one percent oxygen, remaining cells were removed from the media by centrifugation and these conditioned media were immediately used for the experiments. Normoxic and hypoxic cells were used for the migration experiments immediately after completing the oxygen deprivation protocol described in the "Breast cancer cell lines" paragraph.

The cell suspensions were mixed with 100 μl of a buffered collagen solution (pH 7.4), containing 1.67 mg/ml bovine collagen type I (Invitrogen, Cohesion Technologies, Palo Alto, CA). This mixture was filled into self-constructed migration chambers, which consisted of a microscopic glass slide, wax walls on three sides, and a cover slip on top. After polymerization of the collagen at 37°C in a humidified 5% CO_2 _atmosphere, the remaining chamber volumes were filled with the same media that were used for the cell suspensions, and the chambers were sealed on the fourth side with wax. The migration of the cells was recorded by time-lapse videomicroscopy for 1 hour (NG) or for 15 hours (tumor cells) at 37°C. The paths of 30 randomly selected cells were digitized by computer-assisted cell tracking and the part of moving cells was calculated for each time interval, one minute for NG or 15 minute for tumor cells. Inhibition of the non-muscle myosin II activity was achieved using the specific pharmacological inhibitor blebbistatin in all cell lines. Statistically significant changes were calculated from the mean migratory activities in the steady state phase using the unpaired and undirected Student's *t *test. A *P *value lower than 0.05 was considered statistically significant; the *P *value is provided wherever statistical significance was reached.

### Proliferation assay

The proliferation of tumor cells under normoxic and hypoxic conditions was measured by bioreduction of the tretrazolium salt XTT to a formazan derivative with phenazine methosulphate (PMS) as an intermediate electron acceptor [30]. For each condition, ten samples of 5,000 cells in 300 μl medium were seeded per well in a 96-well plate and grown for four days under normoxic conditions or according to the hypoxia protocol described above. After washing with PBS, the cells were incubated for 4 hours at 37°C in 250 μl culture medium without phenol red (PAA), and 50 μl XTT/PMS-solution (freshly prepared mixture of 5 ml XTT (1 mg/ml) and 25 μl PMS (5 mM)). The XTT-formazan derivative was measured at 450 nm using a microplate reader (BioTek Instruments, Bad Friedrichshall, Germany).

### Flow cytometric analysis of cytokine and chemokine release

Flow-cytometry analysis of released signal substances was performed using a fluorescent immunoassay based FlowCytomix kit (Bender MedSystems, Vienna, Austria) according to the manufacturer's protocol. Levels of the following chemokines and cytokines were assessed: G-CSF (granulocyte-colony stimulating factor), IFN-α and -γ (interferon-α and -γ), interleukin-1α, -1β, -2, -4, -5, -6 and -8, IP-10 (IFN-γ inducible protein-10), MCP-1 (monocyte chemoattractant protein), MIG (monokine induced by IFN-γ), MIP-1α and -1β (macrophage inflammatory protein-1α and -1β), NGF (nerve growth factor), RANTES (regulated on activation, normal T-cell expressed and secreted), and TNF-α (tumor necrosis factor -α).

One million cells of each cell line were incubated in 2 ml culture medium as described in the "Breast cancer cell lines" chapter either under normoxic or hypoxic conditions. After completing three days of oxygen deprivation, or the same time under normal culture conditions, the regular culture media was removed and replaced by 1 ml of the media without supplements. After further 24 hour incubation either at one percent oxygen or normoxia, the cell culture supernantants were collected and remaining cells were removed by centrifugation. These supernatants were directly applied to the above described FlowCytomix kit for the investigation of the indicated chemokines and cytokines.

### PCR analysis

The expression of HIF-1α and of its regulated downstream target gene carbonic anhydrase IX (CA IX) was analysed by PCR. Total RNA was isolated from all tumor cell lines using the NucleoSpin RNA II kit (Machery&Nagel, Düren, Germany). An equal amount of RNA of each sample (2 μg) was transcribed into cDNA using a First Strand cDNA Synthesis Kit (Fermentas, St. Leon-Rot, Germany). The resulting cDNA was amplified in 40 cycles. CA IX specific primer sequences were: forward: 5'-GCA GGA GGA TTC CCC CTT G-3'; reverse: 5'-GGA GCC TCA ACA GTA GGT AGA T -3' (annealing temperature 56.1°C; resulting in a 228 base pair product), and HIF-1α specific primer sequences were: forward 5'-GGC GCG AAC GAC AAG AAA AAG-3'; reverse: 5'-CCT TAT CAA GAT GCG AAC TCA CA-3' (annealing temperature 56.65°C; resulting in a 154 base pair product). PCR products were then subjected to gel electrophoresis on a 1.7% agarose gel (Roth, Karlsruhe, Germany) and ethidium bromide staining. A 100 base pair DNA Ladder (Fermentas) was used to control the correct size of the PCR products. Staining intensities were quantified using the ImageJ software (NHI, Bethesda, MD).

## Results

### Migratory activity in response to oxygen deprivation

**Figure 1:**
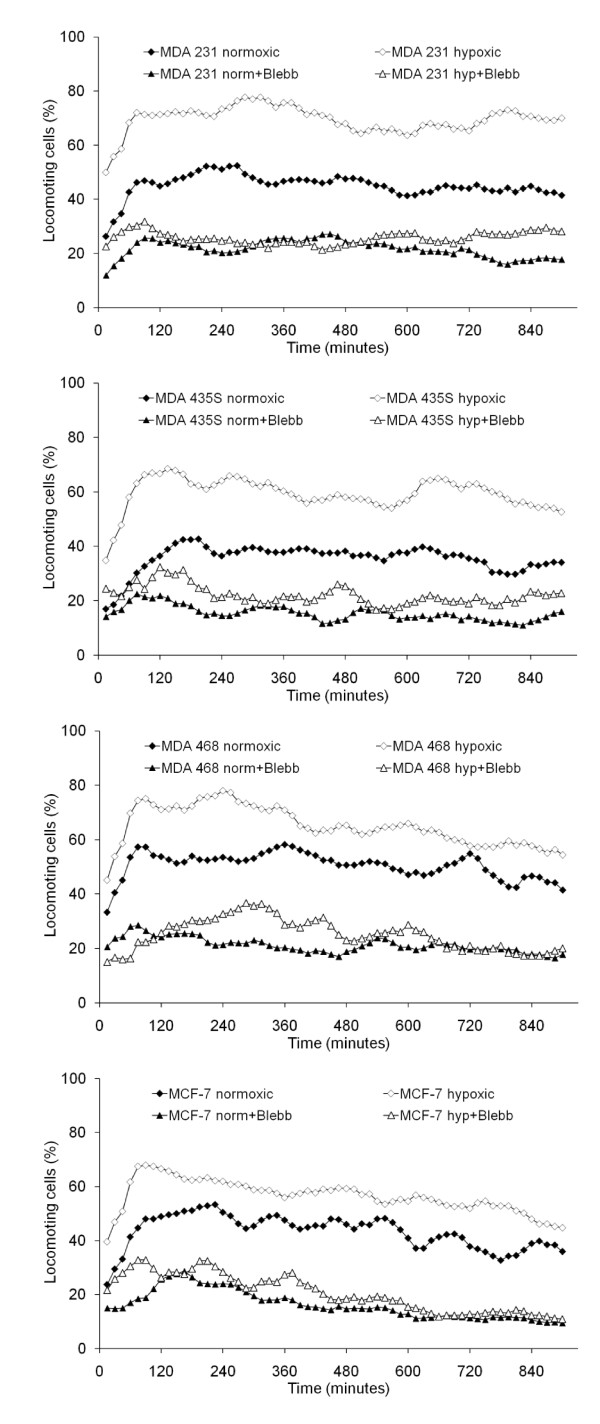
**Migratory activity of tumor cells lines in response to hypoxia**. The graphs show mean values of three independent experiments (90 cells were analyzed per sample). Blebbistatin (Blebb) was used at a concentration of 100 μM.

All of the investigated tumor cells developed spontaneous migratory activity after incorporation within a three-dimensional collagen matrix (Figure 1). This migratory activity increased when the cells were incubated under hypoxic conditions. The migratory activity of MDA-MB-231 cells increased from 44.9 ± 18.0 to 69.4 ± 0.5% locomoting cells, the migratory activity of MDA-MB-435S cells increased from 35.5 ± 9.2 to 59.2 ± 12.2% locomoting cells, the migratory activity of MDA-MB-468 cells increased from 50.4 ± 10.8 to 64.8 ± 9.0% locomoting cells, and the migratory activity of MCF-7 cells increased from 43.1 ± 8.5 to 56.4 ± 6.9% locomoting cells. Although these changes were strong and self-evident, they did not reach statistical significance due to the high standard deviations, whereas the most obvious increases in MDA-MB-231 and MDA-MB-435S cells were close to the significance level (*P *= 0.078 and *P *= 0.055, respectively). However, the migratory activity of both normoxic and hypoxic cells was significantly down-regulated to the same level by inhibition of the non-muscle myosin II activity with the specific pharmacological inhibitor blebbistatin in all cell lines (Figure 1): Down-regulation in normoxic MDA-MB-231 cells to 21.6 ± 3.5% locomoting cells (*P *= 0.048), and hypoxic MDA-MB-231 cells to 25.8 ± 6.8% locomoting cells (*P *= 0.003); down-regulation in normoxic MDA-MB-435S cells to 15.4 ± 6.2% locomoting cells (*P *= 0.034), and hypoxic MDA-MB-435S cells to 22.1 ± 2.5% locomoting cells (*P *= 0.027); down-regulation in normoxic MDA-MB-468 cells to 21.1 ± 3.0% locomoting cells (*P *= 0.003), and hypoxic MDA-MB-468 cells to 25.0 ± 10.6% locomoting cells (*P *= 0.020); down-regulation in normoxic MCF-7 cells to 16.2 ± 5.2% locomoting cells (*P *= 0.003), and hypoxic MCF-7 cells to 20.1 ± 13.4% locomoting cells (*P *= 0.027).

### Induction of tumor cell migration by conditioned medium

**Figure 2:**
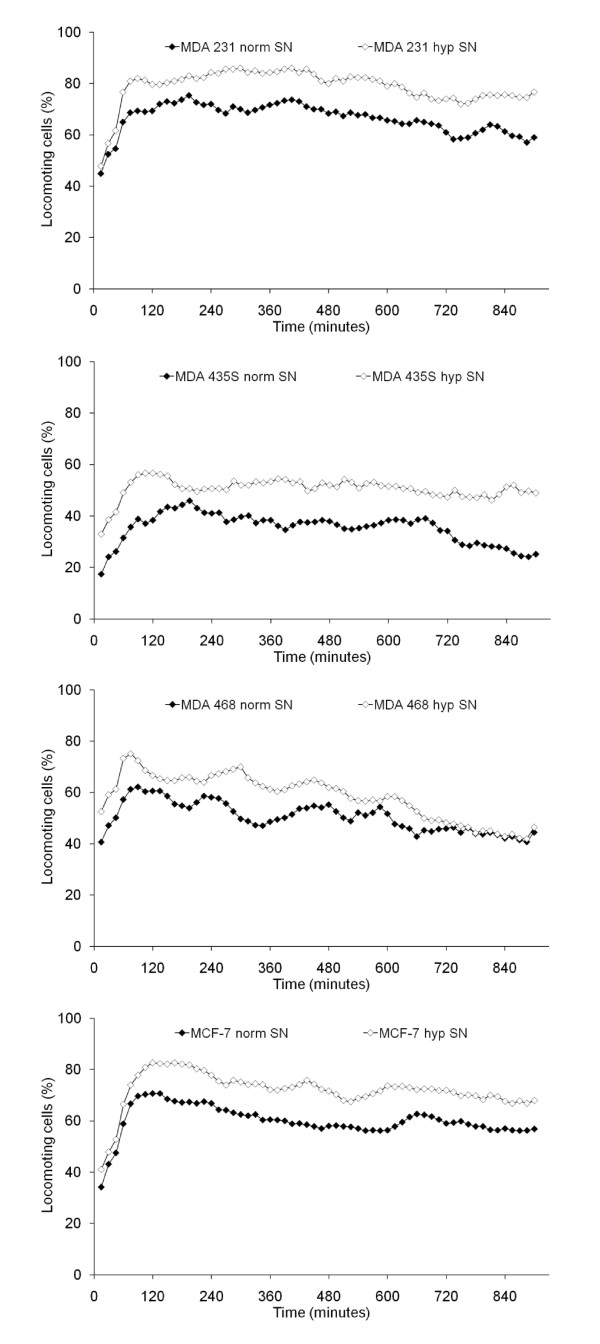
**Influence of cell culture supernatants on the migratory activity of the tumor cell lines**. The graphs show mean values of three independent experiments (90 cells were analyzed per sample). SN = supernatant.

Both normoxic and hypoxic cell culture supernatants strongly stimulated the migratory activity of their respective cell line, with the hypoxic supernatant having a greater effect (Figure 2). The effects of hypoxic supernatant were statistically significant for MDA-MB-231 (*P *= 0.033) and MDA-MB-435S cells (*P *< 0.001). Normoxic and hypoxic cell culture supernatants, respectively, stimulated the migratory activity of MDA-MB-231 cells to 66.1 ± 3.3 and 78.5 ± 0.6% locomoting cells, of MDA-MB-435S cells to 35.2 ± 0.4 and 50.4 ± 3.0% locomoting cells, of MDA-MB-468 cells to 50.3 ± 1.6 and 58.1 ± 12.6% locomoting cells, and of MCF-7 cells to 59.8 ± 9.4 and 71.8 ± 4.5% locomoting cells.

### Release of inflammatory mediators: cytokines and chemokines

**Figure 3:**
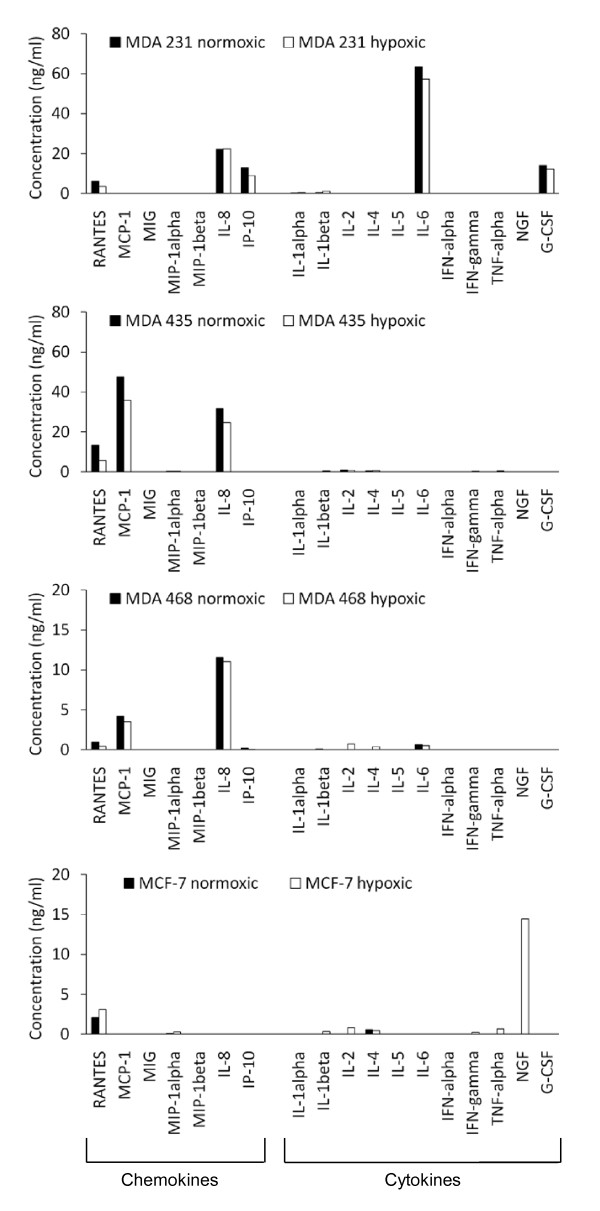
**Release of cytokines and chemokines under normoxic and hypoxic cell culture conditions**. Concentrations are given in ng per ml culture medium, which accounts for 500,000 cells that were initially seeded. Please note, that the scale of the ordinate differs between the cell lines

Expression levels of cytokines and chemokines released from the cell lines under normoxic and hypoxic conditions are shown in Figure 3 (supplemented by Table 2). Generally, the non-luminal cell lines differed significantly to the luminal MCF7 cell line in expressing IL-8 chemokine. The post-EMT cell line MDA-MB-231 differed to the other post-EMT, basal and luminal cell lines in being the only cancer cell line showing IL-6 and G-CSF cytokine and IP-10 chemokine expression. The post-EMT MDA-MB-435 and basal cell line MDA-MB-468 were similar in expressing MCP-1. Only the MCF7 cell line showed G-CSF expression. All cells expressed low levels of RANTES and its expression was increased in hypoxic conditions for the MCF7 cell line. Levels of cytokine and chemokine were different between cell lines cultured in hypoxic and normoxic conditions when expressed.

**Table 2:** Release of substances by the tumor cell lines

Chemokine or cytokine	MDA-231 normoxic	MDA-231 hypoxic	MDA-435 normoxic	MDA-435 hypoxic	MDA-468 normoxic	MDA-468 hypoxic	MCF-7 normoxic	MCF-7 hypoxic
RANTES	6.04	3.37	13.30	5.69	0.94	0.41	2.08	3.10
MCP-1			47.58	35.71	4.18	3.49		
MIG								
MIP-1alpha			0.17	0.27			0.11	0.29
MIP-1beta								
IL-8	22.14	22.41	31.63	24.66	11.57	11.01		
IP-10	12.90	8.90			0.19	0.07		
								
IL-1alpha	0.05	0.30						
IL-1beta	0.38	1.02		0.36	0.12			0.33
IL-2			0.83	0.65		0.75		0.83
IL-4			0.41	0.55		0.38	0.53	0.47
IL-5								
IL-6	63.32	57.23			0.62	0.49		
IFN-alpha								
IFN-gamma				0.14				0.25
TNF-alpha				0.39				0.66
NGF								14.42
G-CSF	13.98	12.17						

Note: This table supplements Figure 3 by providing the actual values (ng/ml).

### Cell proliferation and hypoxia-associated gene expression induced by hypoxic conditions

**Figure 4:**
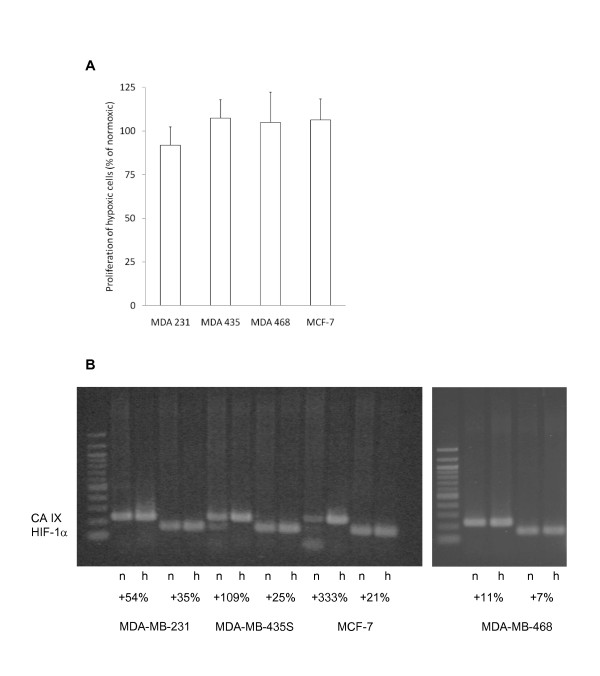
**Growth and HIF-1α/CA IX expression of the tumor cell lines in response to hypoxia**. (A) The growth of tumor cells under hypoxic conditions was compared to the growth under normal (normoxic) cell culture conditions. The graph shows mean values and standard deviations of three independent experiments. (B) The expression of HIF-1α and its target gene CA IX in the tumor cells under normoxic (n) and hypoxic (h) conditions was analysed by PCR. Numbers show the percent increase of staining intensities in hypoxic cells in comparison to normoxic cells. The shown blot holds true for three independently performed analyses with similar results.

In contrast to the migratory activity, tumor cell proliferation was not markedly influenced by oxygen deprivation, as shown by an XTT assay (Figure 4A). In comparison to normal oxygen conditions, hypoxic MDA-MB-231 cells showed a small reduction in proliferation to 91.9 ± 10.5%, MDA-MB-435S cells showed increased proliferation to 107.5 ± 10.5%, as did MDA-MB-468 cells to 105.0 ± 17.2%, and MCF-7 cells to 106.5 ± 11.9% (Figure 4A). None of these changes was statistically significant. These results have confirmatory character with regard to the fact that the amount of released cytokines and chemokines do not vary as a result of distinct proliferation activities.

Gene expression changes were seen for CA IX and HIF-1α in all the cell lines investigated (Figure 4B). RNA levels for CA IX and HIF-1α generally were upregulated by oxygen deprivation. This increase of CA IX expression delivers proof that the herein used protocol for oxygen deprivation causes the well described metabolic switch in the breast cancer cell lines.

### Effect of conditioned medium on NGs

**Figure 5:**
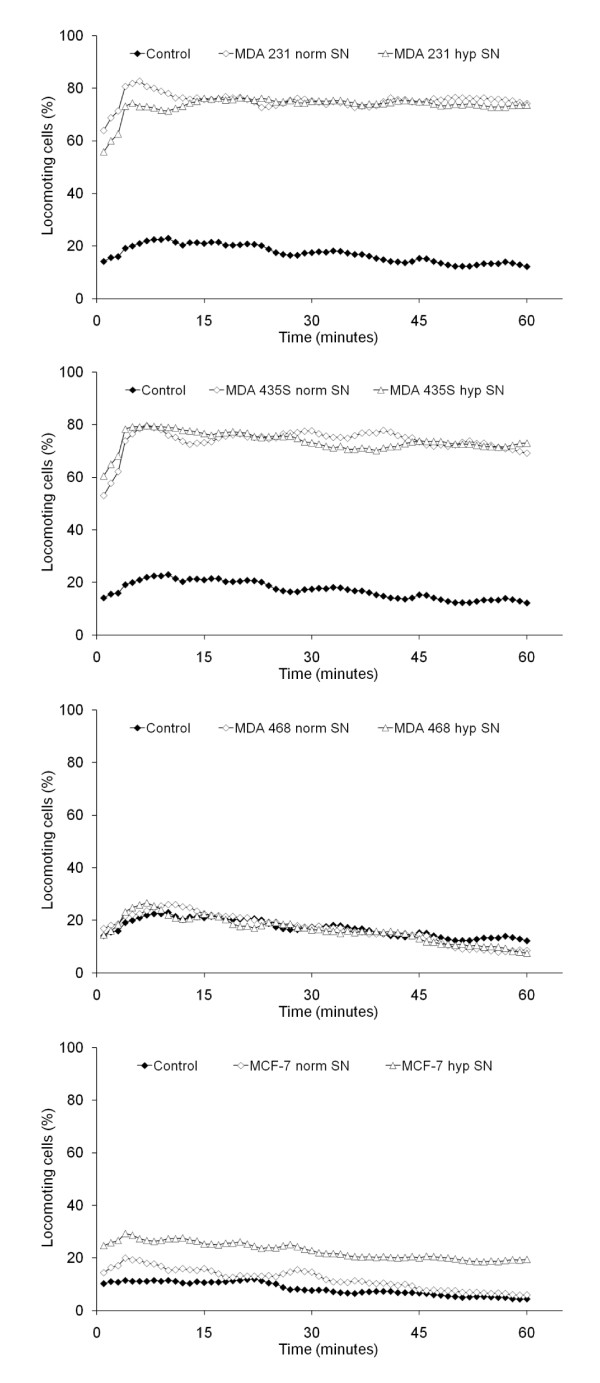
**Response of neutrophil granulocytes to cell culture media conditioned by tumor cells under normoxic (norm) or hypoxic (hyp) conditions**. The graphs show mean values of four independent experiments (120 cells were analyzed per sample). SN = supernatant.

The ability of conditioned medium (CM) to induce migratory activity on NGs was investigated (Figure 5). Conditioned medium from the cell lines that released high amounts of chemokines *i.e*. MDA-MB-231 and MDA-MB-435S cells, significantly stimulated the migratory activity of NGs regardless of whether hypoxic or normoxic culture conditions were used (Figure 5). CM from both normoxic and hypoxic MDA-MB-231 cells increased migratory activity from 17.1 ± 14.5% to 75.2 ± 18.0% (*P *= 0.002) and to 73.4 ± 18.3% (*P *= 0.003), respectively. CM from normoxic and hypoxic MDA-MB-435S cells increased the migratory activity from 17.1 ± 14.5% to 73.7 ± 17.5% (*P *= 0.002) and to 73.8 ± 16.4% (*P *= 0.002), respectively. In contrast, CM from MDA-MB-468 cells had no influence on the migratory activity of NGs, while MCF-7 CM from cells grown under hypoxic conditions only weakly stimulated the migratory activity from 17.1 ± 14.5% to 22.8 ± 15.6% locomoting cells.

The only chemokine that was released by all the non-luminal breast cancer lines, (MDA-MB-231, MDA-MB-435S and MDA 468) was interleukin-8, but blockade of the relevant receptors CXCR1 and CXCR2 with appropriate antibodies only slightly reduced the migratory activity of NG suggesting that other mediators might be involved in their activation (data not shown).

## Discussion

Hypoxia is associated with some forms of high grade breast tumors including basal-like cancers and is associated with poor prognosis [10,11,31,32]. The reason for tumor hypoxia is generally believed to result from tumor cell growth outstripping the rate of angiogenesis [2,3] and consequently, cancer cells react by upregulation of HIF-1α gene and its downstream target genes, including CA IX. One has to keep in mind that experimental normoxic conditions with 21 percent oxygen in cell culture are likely to be higher than *in vivo *oxygen tensions. Although much effort has been made to detect hypoxic regions within tumors, e.g. by positron-emission tomography [33,34], absolute oxygen tensions are difficult to measure. In glial-derived tumors, measurements of 10 to 0.5 precent oxygen were defined as moderate hypoxia, whereas oxygen tensions at 0.1 percent were defined as severe hypoxia [35]. Therefore, oxygen deprivation *in vitro *can only provide results in a comparative manner between cells incubated at different oxygen concentrations, regardless which of the widely varying oxygen deprivation protocols is used.

Our results provide evidence that hypoxia generally increases the migratory activity of breast carcinoma cells but does not appear to influence tumour cell proliferation. This might explain the observation that anti-angiogenic therapeutic treatment can lead to an enhanced metastasis formation [6,8]. Thus, such treatment should be accompanied by anti-metastatic strategies that inhibit tumor cell migration and metastasis formation. A better understanding of the mechanism by which the migratory activity is induced could lead to targeted therapy.

We have reported previously that T24 bladder carcinoma cells stimulate their own migratory activity by the release of interleukin-8 even under normal (non-hypoxic) conditions in an autocrine fashion [36]. Furthermore, several classes of axon guidance molecules have been reported to act in an autocrine fashion in cancer cells, too, inducing invasion and metastasis development [37]. Thus, autocrine activation of tumor cell migration seems to be a frequent phenomenon and is supported by the results in the current study where, even under normoxic conditions, tumor cells release inflammatory mediators that stimulate their own migratory activity.

Importantly, the levels and profile of pro-inflammatory cytokine and chemokine mediators change under hypoxic conditions to strongly enhance migratory activity. However, there is no common signal substance upregulated in all investigated cancer cell lines, which leads to the conclusion that the cytokine or chemokine responsible for the increase in the migratory activity either differs between the various cell lines, or is not among the herein investigated signal substances. Therefore, further studies are needed to characterize this ligand in each cell line, e.g. by knockdown experiments or receptor-blocking antibodies. In the current study, we have shown that the non-luminal breast cancer cell lines including MDA-231, MDA-435 and MDA-468 are all characterised by increased IL-8 production compared to the luminal MCF7 cell line. Previous studies have shown that IL-8 is produced in response to cell stress, including hypoxia [38], and can induce increased cell migration, proliferation, angiogenesis and neutrophil recruitment [39]. Moreover, IL-8 increases metastasis and can cause resistance to chemotherapy [39]. Intriguingly, our data suggests that IL-8 production could be an intrinsic property of the non-luminal cancer phenotype because the post-EMT and basal cell lines investigated here both showed IL-8 production, whereas the luminal cell lines did not. Moreover, we found little or no difference in IL-8 production in response to hypoxia among the non-luminal cancer cell lines. Our data may confirm an involvement of IL-8 in breast cancer cell migration, and support previous studies concerning the role of IL-8 in tumour cell migration [36]. In vitro studies have recently shown that microRNA (miRNA) 17/20 can inhibit migration of post-EMT MDA-MB-231 breast cancer cells by repressing IL-8 [40]. However, these data do not explain the higher migratory activity under hypoxia as compared to normoxia, since IL-8 release does not increase in response to oxygen deprivation. Besides a blockade of the responsible receptors, once the ligands are identified, other possible mechanisms for attenuating cancer cell migration involve inhibition of the cytoskeletal non-muscle myosin II, which is a key mediator of locomotory force generation in the migration of carcinoma cells and leukocytes [15]. Here we provided evidence of the functionality of this pathway by the blockade of non-muscle myosin II in tumor cells using the specific inhibitor blebbistatin, which completely abolished migration in cancer cell lines and in neutrophil granulocytes treated with conditioned medium.

Our observation that hypoxic cells release inflammatory mediators capable of neutrophil recruitment could explain why some basal-like cancers are associated with an extensive inflammatory infiltrate [41,42]. Together with macrophages, neutrophil granulocytes can make up to 50 percent of the tumor mass [43]. Conflicting reports have shown an association between the presence of inflammatory cells, principally CD8^+ ^T lymphocytes, and a role in metastasis formation and clinical outcome [44]. Basal-like medullary cancers are associated with a mixed inflammatory cell pattern and this is generally thought to be due to a host response to focal necrosis that is commonly seen with this tumor type. But, our data suggest that tumour cells might take an active role in recruiting inflammatory cells through production of pro-inflammatory mediators. We showed that not all basal cells have this capability because only conditioned medium from MDA-MB-231 and MDA-MB-435S cells significantly increased the migratory activity of NGs, and that this property is intrinsic, irrespective of hypoxic conditions. Although there seem to be strong similarities between MDA-MB-231 and MDA-MB-435S cells, it is necessary to mention that the provenance of MDA-MB-435S is questionable because it may have evolved from a melanoma cell line [45,46]. More recently, MDA-MB-435 cells have been described as breast cancer cells expressing melanocytic differentiation markers [47]. In contrast to the other basal-like cell lines, conditioned medium released by MDA-MB-468 did not induce neutrophil migratory activity, and medium from hypoxic MCF-7 cells only weakly stimulated neutrophil migratory activity.

Interestingly, although hypoxia led to an increase of HIF1-α and CA IX RNA expression in all the cell lines, our study did not show significant differences in the RNA levels of HIF-1α or CA IX, or a correlation with migratory activity, irrespective of phenotype. A possible explanation for this is due to the *in vitro *cancer cell model used in our study with its absence of fibroblasts. There is increasing evidence showing tumor-stromal interaction is important in influencing tumor aggressiveness. Previous studies have shown that conditioned medium from tumor-derived fibroblasts can promote basal-like properties including EMT [48]. In addition, although basal-like breast cancers have been reported to show a generally worse prognosis than luminal-like cancers [49], the tumor biological grade is of fundamental importance in determining clinical outcome. It has been proposed that hypoxia might induce EMT by an upregulation of TWIST and other EMT-regulators [50,51], contributing to basal-like properties. Our results show, that the release of signal substances by tumor cells does not only influence the tumor-stormal interaction, as described by other groups [48], but has direct autocrine effects, too, painting an even more complex picture on how hypoxia promotes tumor progression.

## Conclusions

The migratory activity of the luminal and non-luminal breast tumour cell lines investigated in this study showed increased migration, mediated in an autocrine fashion, under oxygen deprivation. We found differential patterns of cytokines and chemokines between luminal and non-luminal breast cancer cell lines and changes for some mediators when the cell lines were subjected to hypoxia. Importantly, post-EMT and basal cell lines were characterised by IL-8 production whereas it was absent in the luminal-type MCF7 cell line. Targeting of IL-8 could present a therapeutic strategy for reducing tumour metastasis in breast cancer.

## List of abbreviations

CA: carbonic anhydrase; CK: cytokeratin; CM: conditioned medium; EMT: epithelial-mesenchymal transition; ER: oestrogen receptor; G-CSF: granulocyte-colony stimulating factor; HIF: hypoxia-inducible factor; IFN: interferon; IP: IFN-γ inducible protein; MCP: monocyte chemoattractant protein; MIG: monokine induced by IFN-γ; MIP: macrophage inflammatory protein; NG: neutrophil granulocytes; NGF: nerve growth factor; PGR: progesterone receptor; PMS: phenazine methosulphate; RANTES regulated on activation, normal T-cell expressed and secreted; TNF: tumor necrosis factor

## Competing interests

The authors declare that they have no competing interests.

## Authors' contributions

MJV performed the tumor cell experiments, MFM performed the NG experiments. BN supported the analysis of the cell migration data. MJV, DGP, KSZ and FE designed the study and analyzed the results, MJV, DGP and FE wrote the manuscript and made the figures. All authors have read and approved the final manuscript.

## Pre-publication history

The pre-publication history for this paper can be accessed here:

http://www.biomedcentral.com/1471-2407/11/158/prepub
